# The Oxytocin Receptor Gene (*OXTR*) Variant rs53576 Is Not Related to Emotional Traits or States in Young Adults

**DOI:** 10.3389/fpsyg.2018.02548

**Published:** 2018-12-11

**Authors:** Tamlin S. Conner, Karma G. McFarlane, Maria Choukri, Benjamin C. Riordan, Jayde A. M. Flett, Amanda J. Phipps-Green, Ruth K. Topless, Marilyn E. Merriman, Tony R. Merriman

**Affiliations:** ^1^Department of Psychology, University of Otago, Dunedin, New Zealand; ^2^Ara Institute of Canterbury, Christchurch, New Zealand; ^3^Department of Biochemistry, University of Otago, Dunedin, New Zealand

**Keywords:** oxytocin, genetics, genes, rs53576, emotion, mental health, well-being, mood

## Abstract

**Background:** To understand the genetic underpinnings of emotion, researchers have studied genetic variants in the oxytocin system, a hormone and neurotransmitter important to socio-emotional functioning. The oxytocin receptor gene (*OXTR*) variant rs53576 has been associated with emotional traits such as positive affect and related constructs such as optimism and self-esteem. Individuals carrying the A allele (AG and AA genotypes) of rs53576 have been found to score lower in these traits when compared to GG homozygotes, although not always. Given recent mixed evidence regarding this polymorphism, replication of these associations is critical.

**Methods:** Using a cross-sectional design, the present study tested the association between rs53576 and a wide variety of emotional traits and states in a sample of 611 young adults ages 18 – 25 of various ethnicities (European, Asian, Māori/Pacific Islander, other). Participants completed standard trait measures of positive and negative affect, depressive symptoms, life engagement, psychological well-being, optimism, and self-esteem. They also completed state measures of positive and negative affect and life engagement for 13-days using Internet daily diaries.

**Results:** Controlling for ethnicity and gender, variation at the *OXTR* variant rs53576 obtained from blood samples was not related to any of the emotional traits or states. This null finding occurred despite measuring emotions in “near to real time” using daily diaries and having sufficient power to detect a medium effect size difference between homozygous genotype groups.

**Conclusion:** These findings suggest that variation at the rs53576 locus may not be as involved in emotional differences as initial studies suggested.

## Introduction

Oxytocin is a neurotransmitter and hormone that regulates a variety of social and emotional behaviors including trust and maternal bonding (Campbell, 2010). Oxytocin also plays a role in emotional processes by attenuating the stress response and promoting feelings of calmness and well-being (Kirsch et al., 2005; Uvnäs-Moberg et al., 2005; Campbell, 2010; IsHak et al., 2011). The oxytocin receptor appears to be an important neurochemical mechanism linking oxytocin to emotion (IsHak et al., 2011). Rodent studies show a higher concentration of oxytocin receptors in the central amygdala that inhibit amygdala activity when oxytocin is introduced (Huber et al., 2005). Oxytocin receptors have also been discovered on serotonergic neurons in the raphe nuclei that facilitate serotonin release and may drive anxiolytic effects of oxytocin (Yoshida et al., 2009).

In light of these mechanisms, genetic differences in the oxytocin receptor gene (*OXTR*) may influence emotional differences between people. One previously studied variant of *OXTR* is rs53576, a single nucleotide polymorphism located in intron 3 (Wu et al., 2005). The A (minor) allele of rs53576 has been associated with impaired social and maternal behaviors (e.g., Rodrigues et al., 2009; Kogan et al., 2011; Riem et al., 2011). Similarly, the G allele of rs53576 has been associated with greater sociality in people (Li et al., 2015). However, evidence for the association between rs53576 and emotional traits is inconsistent.

Several studies have tested for associations between rs53576 and emotional traits. A study of 289 German adults found that men with the AA genotype reported lower trait positive affect compared to men with the AG or GG genotypes; however, only 13 men had the AA genotype so results should be interpreted cautiously (Lucht et al., 2009). Saphire-Bernstein et al. (2011) found that among a sample of 344 mixed-ethnicity adults, both men and women carriers of the A allele (AA, AG) reported lower trait optimism, self-esteem, and mastery, as well as higher self-reported depression compared to GGs. But a larger sample of 1229 Caucasian women showed no such association between rs53576 and optimism (Cornelis et al., 2012). Moreover, an earlier case control study (92 cases/ 192 controls) found that unipolar depression was more likely among patients with the GG genotype of rs53576 – not the AA or AG genotype (Costa et al., 2009), which was replicated in another recent case control study (Costa et al., 2017). Similarly, a large population based study (*n* = 1185) found AA men had *lower* depression symptoms (vs. GG or GA), although this genotype was not overly represented among suicide victims (*n* = 763) (Wasilewska et al., 2017). Finally, a meta-analysis reported that rs53576 was unrelated to depression but was related to elevated general sociality for the GG genotype (Li et al., 2015). Taken together, these studies paint a mixed picture of the relationship between rs53576 and emotion.

Given the high likelihood for false-positives in genetic association studies, it is important to replicate genetic associations such as the *OXTR* rs53576 and emotion, hence our aim here. We measured a variety of emotion constructs at the trait level (positive and negative affect, depressive symptoms, life engagement, psychological well-being, optimism, and self-esteem) and state level (positive and negative affect, life satisfaction) using daily diaries in light of evidence that “near to real time” emotion reports may be especially sensitive for detecting genetic association with emotion (Conner and Barrett, 2012; Finan et al., 2012).

## Materials and Methods

### Participants and Procedure

Participants were 611 young adults studying at the University of Otago, New Zealand (see Table 1, demographics). The data were collected in 2011 and 2012 as part of the Daily Life Study, and carried out in accordance with the recommendations and approval of the University of Otago Human Ethics Committee (10/777). All participants gave written informed consent in accordance with the Declaration of Helsinki. Participants completed a baseline survey with demographic questions and trait measures in private cubicles, completed an Internet daily diary survey each day between 3 and 8 pm for 13 consecutive days, gave a non-fasting 22 ml venous blood sample at an on-campus clinic (4 people gave a saliva sample), and completed a final survey.

**Table 1:** Sample demographics and descriptive statistics for emotion measures.

	N (%)	Mean (*SD*)	Observed range	Scale range
Total sample size	611			
Gender				
Female	379 (62.0)			
Male	232 (38.0)			
Ethnicity				
European	487 (79.7)			
Asian	59 (9.7)			
Māori / Pacific Islander	19 (3.1)			
Indian	17 (2.8)			
Mixed ethnicity	10 (1.6)			
Middle Eastern	7 (1.1)			
Other	12 (2.0)			
Sample recruitment				
Psychology classes	537 (87.9)			
Campus employment agency	74 (12.1)			
Age		19.49 (1.54)	17–25	
Daily surveys (# completed)		12 (1)	7–13	0 - 13

	**α^†^**	**Mean (*SD*)**	**Observed range**	**Scale range**

**Trait emotion measures**				
Positive affect	0.843	3.26 (0.55)	1.44–4.67	1–5
Negative affect	0.856	1.85 (0.56)	1.00–4.00	1–5
Depressive symptoms	0.875	14.07 (8.49)	0.00–46.0	0–60
Life engagement	0.780	3.91 (0.58)	1.83–5.00	1–5
Psychological well-being	0.793	3.79 (0.44)	2.33–4.89	1–5
Optimism	0.818	4.68 (1.01)	1.33–7.00	1–7
Self-esteem	0.877	3.71 (0.67)	1.70–5.00	1–5
**State emotion measures^††^**				
State positive affect	0.818	2.98 (0.49)	1.53–4.74	1–5
State negative affect	0.783	1.70 (0.48)	1.01 – 4.02	1–5
Life engagement	0.735	3.32 (0.51)	1.14–4.85	1–5

*^†^Reliability estimates reflect Cronbach’s alphas (α) for trait measures and within-person reliabilities computed on nested data for state measures as recommended by Nezlek (2012). ^††^Aggregated across daily diaries.*

### Measures

The baseline survey measured trait *positive affect* and *negative affect* using an affective circumplex measure (Barrett and Russell, 1999) in which participants rated nine positive affect items (enthusiastic, excited, energetic, happy, cheerful, pleased, calm, content, relaxed) and nine negative affect items (irritable, angry, hostile, nervous, anxious, tense, dejected, sad, unhappy) for how they “generally feel” from 1 (not at all) to 5 (extremely). *Depressive symptoms* were measured using the 20-item Center for Epidemiological Studies Depression Scale (Radloff, 1977) with items answered “in the last week” on a 4-point scale from 0 (never or rarely a symptom occurs) to 3 (most or all of the time it occurs), and *optimism* using the 6-item Life Orientation Test – Revised (Carver et al., 2010) with items answered on a 7-point scale from 1 (strongly disagree) to 7 (strongly agree). The final survey measured *psychological well-being* (18-item Psychological Well-being Scale; Ryff and Keyes, 1995), *life engagement* (6-item Life Engagement Test; Scheier et al., 2006), and *self-esteem* (10-item Rosenberg Self-Esteem Scale; Rosenberg, 1965) with all items answered on 5-point scales from 1 (not at all) to 5 (extremely). The daily diary survey measured *state positive affect* and *state negative affect* using the circumplex scale as above and *state life engagement* using the Life Engagement Test as above, with items rated for how they felt “today” on a scale from 1 (not at all) to 5 (extremely).

### Genotyping

Genotypes for the *OXTR* variant rs53576 were obtained from blood (*n* = 607) or saliva samples (*n* = 4). DNA was extracted from peripheral white blood cells using a guanidine-HCl-based procedure with chloroform extraction (McKinney et al., 2010) and genotyped for the rs53576 genotype (GG, AG, or AA) using a Life Technologies TaqMan 5 nuclease assay (probe id C_3290335_10) and performed according to manufacturer’s instructions. Fourteen percent of samples were repeated with 100% concordance in genotype.

### Statistical Analyses and Power

Analysis of covariance (ANCOVA) tested for genotype differences in each emotion measure while controlling for ethnicity and gender. The three genotype groups (GG, GA, and AA) served as the primary predictor. Ethnicity was coded using a set of three dummy variables (European as reference group 000, compared with Asian 100, Māori/Pacific Islander 010, and Other 001). Gender was coded 0 for men and 1 for women. Gender differences in genotype effects were tested in follow up analyses by modeling the gender x genotype interaction and also by running the analyses for men and women separately. The *p*-value was set to less than 0.005 using a Bonferroni correction (0.05/10 primary tests) to adjust for multiple hypothesis testing. Based on an estimated sample prevalence of GG and AA (43 and 15%) (estimated from Lucht et al., 2009; and Saphire-Bernstein et al., 2011), at least 480 participants were needed to have 0.8 power to detect a medium size effect (*d* = 0.5) between GG and AA at α = 0.005. We found a lower prevalence of AAs in our sample compared to initial estimates, but with a final sample of 611 we still had 0.84 power to detect a medium size difference between the homozygotes at α = 0.005.

## Results

The genotype distributions for the *OXTR* rs53576 variant were GG 275 (45%), AG 262 (42.9%), and AA 74 (12.1%), which were in Hardy Weinberg Equilibrium (*χ^2^* = 0.90, *p* = 0.344). The AA genotype was the least common of the three genotypes, reflecting a frequency *f*(A) of 0.336 for the A allele. The A allele was more common among Asian participants [*f*(A) = 0.610] than European participants [*f*(A) = 0.308, *χ^2^* = 50.86, *p* < 0.001], Māori/Pacific Island participants [*f*(A) = 0.317, *χ^2^* = 14.02, *p* = 0.001] or other ethnicities [*f*(A) = 0.283, *χ^2^* = 22.43, *p* < 0.001].

Table 2 presents the emotion results. There was no evidence of genotype differences in any of the emotion measures that exceeded the Bonferroni adjustment of *p* < 0.005. There were no significant gender × genotype interactions, although when analyzing men and women separately, AA women reported higher self-esteem (*M*_adjusted_ = 3.89) than AG or GG women [AG, *M*_adjusted_ = 3.59; GG, *M*_adjusted_ = 3.60; *F*(*df*) = 3.189, *p* = 0.042], but this association did not exceed the Bonferroni adjustment of *p* < 0.005.

**Table 2:** Emotional Traits and States for each *OXTR* rs53576 Genotype: Raw Means, Standard Deviations, and ANCOVA Results.

		Raw means (*SD*s)				Adjusted means^†^					
		GG *n* = 275	AG *n* = 262	AA *n* = 74		GG	AG	AA		*F (df)*	*P*
**Trait emotion measures**											
Positive affect		3.26 (0.53)	3.26 (0.57)	3.28 (0.55)		3.26	3.26	3.29		0.122 (2)	0.885
Negative affect		1.88 (0.56)	1.86 (0.57)	1.72 (0.49)		1.88	1.86	1.72		2.242 (2)	0.107
Depressive symptoms		14.12 (8.51)	14.18 (8.58)	13.51 (8.15)		14.3	14.1	13.2		0.404 (2)	0.668
Life engagement		3.89 (0.59)	3.91 (0.59)	3.96 (0.55)		3.88	3.92	3.99		0.936 (2)	0.393
Psychological well-being		3.78 (0.44)	3.78 (0.45)	3.81 (0.44)		3.77	3.79	3.84		0.596 (2)	0.551
Optimism		4.69 (1.02)	4.71 (1.04)	4.60 (0.87)		4.68	4.71	4.63		0.217 (2)	0.805
Self-esteem		3.69 (0.65)	3.70 (0.71)	3.83 (0.59)		3.68	3.71	3.84		1.483 (2)	0.228
**State emotion measures**											
Positive affect		2.99 (0.51)	2.96 (0.47)	3.05 (0.51)		2.98	2.96	3.08		1.581 (2)	0.207
Negative affect		1.73 (0.50)	1.68 (0.48)	1.68 (0.45)		1.73	1.68	1.67		0.859 (2)	0.424
Life engagement		3.29 (0.51)	3.32 (0.52)	3.46 (0.48)		3.28	3.32	3.47		3.492 (2)	0.031

*SD, standard deviations are in parentheses under raw means. ^†^Means adjusted for ethnicity and gender in analysis of covariance (ANCOVA). The F and p-values were based on the adjusted means.*

## Discussion

Variation at the *OXTR* rs53576 locus was not associated with self-reported emotion in our sample. Even with data gathered in real time, we still found no evidence of genetic association. The present findings are similar to the null findings from Li et al’s. (2015) meta-analysis and Cornelis et al. (2012) who found no association between rs53576 and optimism, but contrast with findings from Lucht et al. (2009) who found lower trait positive affect in AA men, and with Saphire-Bernstein et al. (2011) who found lower optimism, mastery, self-esteem and depression in A carriers. We do not know what factors account for these differences, but sample characteristics including ethnic ancestry and environmental factors could play a role. Our sample, like that of Cornelis et al. (2012), consisted mainly of European participants who have a lower frequency of the A allele e.g., [*f*(A) = 0.336 in our sample], whereas the Saphire-Bernstein et al. (2011) sample included more Asian participants who have a higher frequency of the A allele [*f*(A) = 0.624]. However, ethnicity differences may not fully explain the differences because Saphire-Bernstein et al. (2011) controlled for ethnicity in secondary analyses and found the genetic association was reduced but not eliminated. Cornelis et al. (2012) suggested that associations might be more evident in younger rather than middle aged samples; however, our sample was young, ages 18 to 25 (mean age 19).

Main effect analyses could mask gene-environment and gene-gene interactions, such that the expression of rs53576 may be conditional upon life stress or cultural norms (Kim et al., 2011; Sasaki et al., 2011; McQuaid et al., 2013; LeClair et al., 2016) or conditional upon other genotypes. Sample differences in stress could possibly interact with rs53576 to produce variability in results. Participants from Saphire-Bernstein et al. (2011) were recruited for a study of stress and coping and could be at higher stress levels than our participants, which could accentuate genotype effects. However, participants in the Nurses’ Health Study had moderate levels of stress (Feskanich et al., 2002) yet showed no genotype differences in emotion as a function of rs53576 (Cornelis et al., 2012). It is notable that the largest studies of rs53576 and emotion to date – Cornelis et al. (2012); Wasilewska et al. (2017), and ours – showed either null results or mixed results. Larger sample sizes may have reduced the likelihood of false-positives (Ioannidis et al., 2001; Pickrell et al., 2011).

Genetic variants with demonstrable effects on brain processes are stronger candidates; however, in the case of *OXTR* rs53576, we can find no direct functional evidence of how variation at the rs53576 locus influences processes or oxytocin expression in the brain. Only associative evidence exists. For example, neuroimaging research has found significant brain differences associated with rs53576 (e.g., reduced volume in hypothalamic gray matter and reduced volume and functional connectivity in the amygdala for A alleles by Tost et al., 2010; reduced amygdala volume and reduced connectivity for female A alleles by Wang et al., 2014). However, as noted by Tost et al. (2010), this evidence does not directly support a role for the oxytocin receptor. It is only correlational and therefore it cannot be ruled out that other variants in linkage disequilibrium may actually be driving observed associations. Possible candidates include another *OXTR* variant or a variant of the vasopressin gene neighboring *OXTR* (Gimpl and Fahrenholz, 2001).

We encourage replication in larger samples with greater power to detect small effect sizes. We also encourage future exploration of gene-environment and gene-gene interactions (e.g., Windle and Mrug, 2015). However, the present null findings, coupled with the null or mixed results of prior work and the lack of functional evidence for rs53576 in relation to emotion, suggest that future research on emotion should consider other variants besides rs53576 that may influence the emotional pathways of oxytocin such as rs6449182 in the *CD38* gene, which regulates oxytocin release (Jin et al., 2007), and has been linked to emotional bonding in couples (Algoe and Way, 2014).

## Author Contributions

TC and KM conceived the research. KM, MC, BR, and JF collected the data. AP-G, RT, MM, and TM processed the blood samples and genotyped the DNA and saliva samples. TC and KM wrote the article with assistance from JF, BR, MC, and TM.

## Conflict of Interest Statement

The authors declare that the research was conducted in the absence of any commercial or financial relationships that could be construed as a potential conflict of interest.
